# Targeting Tumor Microenvironment Akt Signaling Represents a Potential Therapeutic Strategy for Aggressive Thyroid Cancer

**DOI:** 10.3390/ijms24065471

**Published:** 2023-03-13

**Authors:** Saied Mirshahidi, Isabella J. Yuan, Alfred Simental, Steve C. Lee, Nathaniel R. Peterson, Pedro A. Andrade Filho, Thomas Murry, Penelope Duerksen-Hughes, Xiangpeng Yuan

**Affiliations:** 1 Department of Basic Sciences, School of Medicine, Loma Linda University, Loma Linda, CA 92354, USA; 2 Cancer Center Biospecimen Laboratory, Loma Linda University Medical Center, Loma Linda, CA 92354, USA; 3 Department of Otolaryngology-Head and Neck Surgery, Loma Linda University Medical Center, Loma Linda, CA 92354, USA

**Keywords:** Akt signaling, cancer stem cell, thyroid cancer, tumor stromal cell, targeting aggressive disease

## Abstract

Effects of the tumor microenvironment (TME) stromal cells on progression in thyroid cancer are largely unexplored. Elucidating the effects and underlying mechanisms may facilitate the development of targeting therapy for aggressive cases of this disease. In this study, we investigated the impact of TME stromal cells on cancer stem-like cells (CSCs) in patient-relevant contexts where applying in vitro assays and xenograft models uncovered contributions of TME stromal cells to thyroid cancer progression. We found that TME stromal cells can enhance CSC self-renewal and invasiveness mainly via the phosphatidylinositol 3-kinase (PI3K)/protein kinase B (Akt) pathway. The disruption of Akt signaling could diminish the impact of TME stromal cells on CSC aggressiveness in vitro and reduce CSC tumorigenesis and metastasis in xenografts. Notably, disrupting Akt signaling did not cause detectable alterations in tumor histology and gene expression of major stromal components while it produced therapeutic benefits. In addition, using a clinical cohort, we discovered that papillary thyroid carcinomas with lymph node metastasis are more likely to have elevated Akt signaling compared with the ones without metastasis, suggesting the relevance of Akt-targeting. Overall, our results identify PI3K/Akt pathway-engaged contributions of TME stromal cells to thyroid tumor disease progression, illuminating TME Akt signaling as a therapeutic target in aggressive thyroid cancer.

## 1. Introduction

Thyroid cancer is the most common malignancy in the endocrine system with continuously increasing incidence and is predicted to become one of the top five most commonly diagnosed cancers by 2030 [1]. The genetic alteration of BRAF^V600E^ is most commonly observed in thyroid cancer, in particular papillary thyroid carcinoma (PTC), and is associated with the disease displaying an aggressive process [2,3,4,5,6,7]. In addition to the BRAF mutation, poorly differentiated (PDTCs) and anaplastic thyroid cancers (ATCs) often have other genetic and/or non-genetic alterations that can lead to the activation of certain signaling pathways involved in cancer cell growth and tumor progression. Activations of such signaling in cancer cells often cause unresponsiveness to treatments commonly used for thyroid cancer, including radiation and chemotherapy [8,9,10,11,12,13,14,15,16]. Currently, the success of targeted therapeutic strategies for advanced thyroid cancers is still limited [17,18,19,20,21,22,23,24,25,26]. The increasing thyroid cancer incidences, combined with the relative lack of effective therapies in treating advanced disease, underscore the need for the identification of mechanisms underlying thyroid cancer progression and for the development of novel therapeutics targeting aggressive disease.

The tumor microenvironment (TME) is comprised of various cellular and non-cellular components that coordinate to promote tumorigenesis and metastasize into a variety of solid malignancies [27,28,29,30,31,32,33,34,35,36]. Tumor cells are capable of inducing the migration of non-malignant cells, such as fibroblasts, immune cells, and endothelial cells, to the TME through paracrine as well as direct cell–cell contact mechanisms, which collectively lead to the development of a tumor-cell-supporting niche [37,38,39,40,41]. Such niches can facilitate the function of cancer stem-like cells (CSCs) [42,43,44,45,46,47], the driving force of tumor initiation and progression [48,49,50]. The influence of tumor stroma on tumor progression is recognized in many types of cancer [27,51,52]. However, the underlying mechanisms by which tumor stromal cells exerting their effects to influence thyroid cancer progression, especially effecting CSCs, remain largely undefined.

A comprehensive understanding of tumor stromal cells’ roles in thyroid cancer TME and the candidate signaling pathways mediating tumor stromal cells’ effects on thyroid CSCs can greatly facilitate the development of new therapies for targeting tumor progression. To identify potential effects and underlying mechanisms of stromal cells affecting thyroid cancer progression, a patient-specimen-derived microenvironment culture system and patient-derived xenograft (PDX) models were used to dissect factors that influence thyroid CSC behaviors, including tumorigenic and metastatic activity. We found that thyroid cancer stromal cells can promote CSC self-renewal and invasion, and the promoting effect is mainly mediated by the PI3K/Akt signaling pathway. Inhibiting Akt activity diminished the influence of tumor stromal cells on CSC cellular behaviors in vitro, and reduced CSC tumorigenesis and metastasis in xenograft models. In a clinical PTC cohort, we found Akt signaling upregulation in patients who had aggressive diseases. Based on our findings, it is implicated that TME stromal cells can potentiate thyroid cancer progression via PI3K/Akt pathway. Targeting TME Akt signaling may represent an efficacious therapeutic strategy for aggressive thyroid carcinoma.

## 2. Results

### 2.1. Tumor Microenvironment Stromal Cells Demonstrated the Capability of Promoting CSC Growth and Invasion

**Figure 1:**
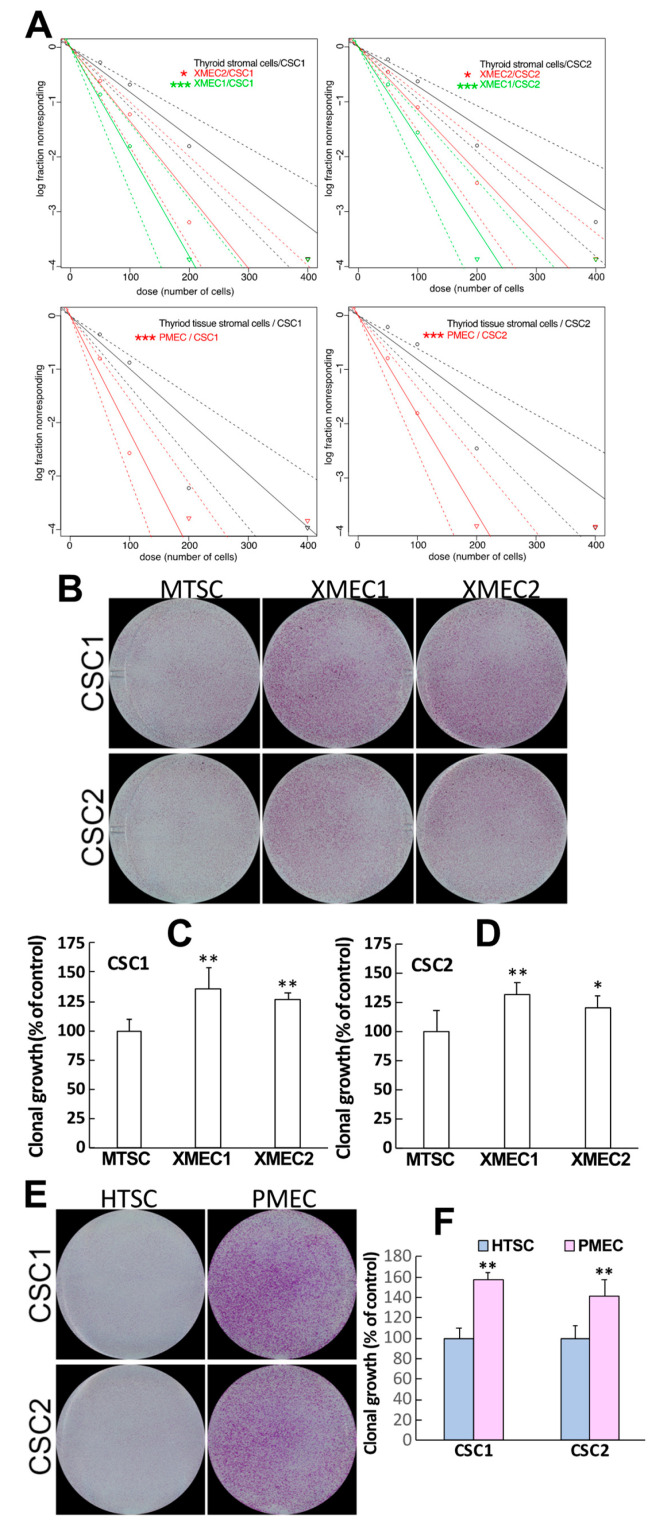
PTC tumor stromal cells enhanced CSC self-renewal and clonal growth. (**A**) Tumor stromal cell increased CSC sphere-formation efficiencies in transwell cocultures as the stromal cells isolated from either patient-derived xenograft tumors (top panel) or patient primary tumors (bottom panel). (**B**–**F**) Tumor stromal cells enhanced CSC clonal growth in transwell cocultures as the stromal cells isolated from either patient-derived xenograft tumors (**B**–**D**) or patient primary tumors (**E**,**F**). Data are representative of at least three independent experiments and shown as mean ± SEM. Mouse normal thyroid stromal cell culture (MTSC), xenograft tumor microenvironment stromal cell culture (XMEC), patient non-tumor thyroid stromal cell culture (HTSC), patient primary tumor microenvironment stromal cell culture (PMEC); * *p* < 0.05, ** *p* < 0.01, *** *p* < 0.001.

It has been demonstrated that CSCs represent the driving force of tumor initiation and progression [53,54,55,56,57,58], while the influence of TME stromal cells on PTC CSC cellular behaviors remained largely to be elucidated. To investigate how tumor stromal cell influence CSC behaviors in PTC microenvironment, we established a transwell coculture system in which tumor stromal cells derived directly from tumor tissues were cocultured in a transwell setting with CSCs isolated from patient specimens. Such transwell cocultures provided conditions that allowed tumor stromal cells to exert their potential influence on CSCs without compromising the downstream analysis of CSC’s biological behaviors. Within the transwell coculture, tumor stromal cells derived from PDX tumors were seeded in the top chambers, and CSCs isolated from clinical PTC specimens were plated in the bottom chambers. With the transwell cocultures, CSCs were maintained in an environment that mimicked in vivo TME, and CSC cellular behaviors were assessed within the microenvironment. The stromal cells derived from PDX tumors demonstrated the capability to promote CSC sphere formation in the transwell cocultures as compared with mouse normal thyroid tissue-derived stromal cells (Figure 1A, upper panel). Importantly, the stromal cells derived from xenograft tumors that were initiated by either CSC1 or CSC2 cell transplantation showed effects of enhancing the sphere-formation efficiencies of both CSC1 and CSC2 cells (Figure 1A, upper panel). To extend these observations into more clinically relevant settings, we subsequently used patient primary-tumor-derived stromal cells replacing xenograft tumor stromal cells in the sphere-formation assays. We found that the stromal cells derived from a PTC patient primary tumor also showed the capability to enhance both CSC1 and CSC2 sphere formation as compared to the patient’s non-tumor thyroid tissue-derived stromal cells (Figure 1A, lower panel). Interestingly, the patient primary tumor stromal cells had a higher efficacy of promoting CSC sphere formation than did the xenograft tumor stromal cells (The sphere-formation efficiencies in patient primary tumor stromal cell coculture were 1.7–3.2 and 1.4–2.9 times of that in PDX tumor stromal cell coculture for the CSC1 and CSC2, respectively).

Next, we investigated whether the stromal cells in the transwell cocultures have influences on the potential of CSC clonal growth. To perform this analysis, the bottom chambers of the transwell system were culture-treated to facilitate cell attachment and clonal growth. We observed that tumor stromal cells were able to enhance CSC clonal growth within the transwell coculture as compared with the controls (Figure 1B). Although tumor stromal cells derived from different tumor specimens showed variable capacities in enhancing CSC clonal growth, either PDX or patient primary tumor stromal cells were capable of augmenting the clonal growth of all the CSC samples tested (Figure 1B–F). To validate the above findings, we performed a set of additional experiments by seeding either CSCs or only culture medium alone within the top chambers and seeding CSCs within the bottom chambers of the transwell cocultures. The sphere formation and clonal growth were analyzed for the CSCs located in the bottom chambers. These experiments were intent on finding whether any cancer cells that might potentially have contaminated the isolated stromal cells could be confounding factors contributing to the results obtained above. There were no differences identified in the bottom chamber CSC’s sphere formation and clonal growth between the conditions with top chamber containing CSCs and top chamber containing medium alone. These results confirmed that the enhanced CSC’s sphere formation and clonal growth in the transwell cocultures were due to tumor stromal cell contribution.

**Figure 2:**
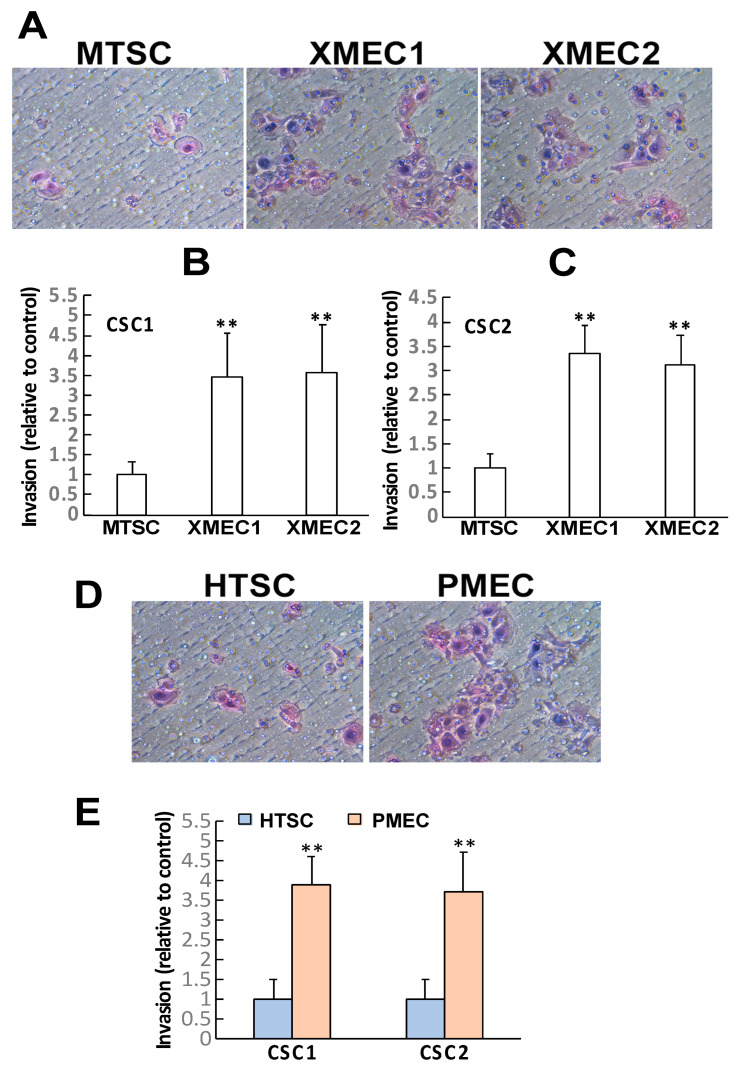
PTC tumor stromal cells enhanced CSC invasiveness. (**A**) Patient-derived xenograft tumor stromal cells increased CSC1 invasive activities in Boyden chamber invasion assays. (**B**,**C**) Quantitative analyses showed different xenograft tumor stromal cells having the capacity to enhance both CSC1 and CSC2 cell invasion. (**D**) Patient primary tumor stromal cells increased CSC1 cell invasive activities. (**E**) Quantitative analyses revealed patient primary tumor stromal cells having the capacity to enhance both CSC1 and CSC2 cell invasion. Data are representative of at least three independent experiments and shown as mean ± SEM. Mouse normal thyroid stromal cell culture (MTSC), xenograft tumor microenvironment stromal cell culture (XMEC), patient non-tumor thyroid stromal cell culture (HTSC), patient primary tumor microenvironment stromal cell culture (PMEC); ** *p* < 0.01. Scale bars: 100 µm.

As tumor cell invasiveness is an essential feature contributing to cancer disease progression and metastasis, we therefore investigated whether tumor stromal cells have any effects on the activity of CSC invasion. We performed Boyden chamber invasion assays in which tumor stromal cells or thyroid tissue stromal cells were seeded in the bottom wells while CSCs were plated in the top insert wells. CSCs that migrated through the Matrigel-matrix, separating the top from the bottom wells, were recorded to indicate CSC’s invasive activities. Enhanced invasive activities were observed with PDX tumor stromal cells seeded in the bottom wells as compared to the mouse normal thyroid tissue stromal cells (Figure 2A, Supplementary Figure S1A). The tumor stromal cells isolated from both CSC1- and CSC2-derived xenografts exhibited the capability of enhancing either CSC1 or CSC2 cell invasion (Figure 2A–C, Supplementary Figure S1A). In addition, patient primary-tumor-derived stromal cells were able to induce enhancements in the invasion of both CSC1 and CSC2 cells compared with patient non-tumor thyroid tissue-derived stromal cells (Figure 2D,E, Supplementary Figure S1B). Interestingly, the stromal cells of patient primary tumors demonstrated higher efficacy in enhancing CSC invasion than did the stromal cells of PDX tumors. Altogether, our results demonstrated that the tumor stromal cells of PTC had the capacity to promote CSC sphere formation and clonal growth, as well as CSC invasiveness.

### 2.2. Tumor Stromal Cells Were Capable of Enhancing JAK/STAT3 and PI3K/Akt Signaling in CSCs

**Figure 3:**
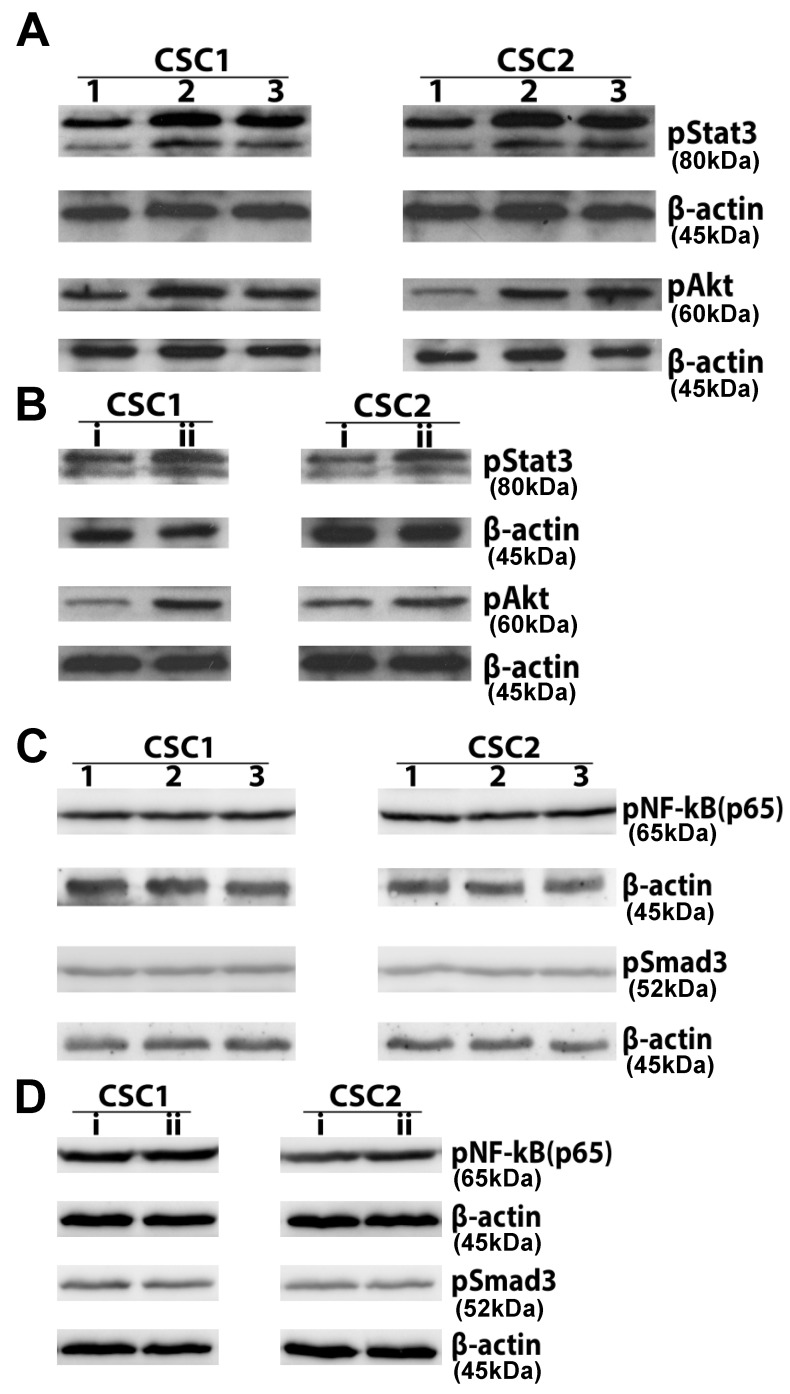
Tumor stromal cells increased PI3K/Akt and JAK/STAT3 signaling in CSCs. (**A**) Xenograft tumor stromal cells enhanced the expression of pStat3 and pAkt in CSCs. (**B**) Patient primary tumor stromal cells enhanced the expression of pStat3 and pAkt. (**C**) Xenograft tumor stromal cells did not affect NF-kB and TGF-β signaling as no alterations were detected in the expression of pNF-kB and pSmad3 in CSCs. (**D**) Patient primary tumor stromal cells did not alter the expression of pNF-kB and pSmad3. Lane 1: CSCs cocultured with MTSC; lane 2: CSCs cocultured with XMEC1; lane 3: CSCs cocultured with XMEC2; lane i: CSCs cocultured with HTSC; lane ii: CSCs cocultured with PMEC. Mouse normal thyroid stromal cell culture (MTSC), xenograft tumor microenvironment stromal cell culture (XMEC), patient non-tumor thyroid stromal cell culture (HTSC), patient primary tumor microenvironment stromal cell culture (PMEC); phospho-Stat3 (pStat3), phospho-Akt (pAkt), phospho-NF-kB p65 (pNF-kB), phospho-Smad3 (pSmad3).

The above observations revealed the capacity of tumor stromal cells to affect CSC behaviors. We next examined the potential signaling pathways that might mediate the effects of tumor stromal cells on CSCs. According to the findings in recent studies, there are several signaling pathways, including JAK/STAT [59,60,61,62,63], PI3K/Akt [64,65,66,67,68,69,70], NF-kB [71,72,73,74,75,76], and TGF-β signaling [77,78,79,80,81,82,83], that could possibly be mediating the stromal cell functioning in PTC, as these signaling pathways have been found to be involved in the effects of stromal cells on tumor cells [59,60,61,62,63,77,78,79,80,81,82,83]. To unveil the potentially engaged signaling pathways, we examined the expression of specific molecules that indicate different signaling pathway activities with Western blotting analyses. It was observed that phosphorylated (activated) STAT3 expression, the indication of JAK/STAT3 signaling activity, was elevated in the CSCs when the cells were transwell cocultured with PDX tumor stromal cells in comparation with mouse normal thyroid tissue stromal cells (Figure 3A). In addition, transwell coculture with patient primary tumor stromal cells similarly increased the pSTAT3 expression in CSCs compared to the coculture with patient non-tumor thyroid tissue stromal cells (Figure 3B). It was also revealed that the expression of phosphorylated Akt, an indication of the PI3K/Akt pathway activity, was increased in CSCs that were transwell cocultured with PDX or patient primary tumor stromal cells as compared with the controls in which the CSCs were transwell cocultured with mouse normal thyroid or patient non-tumor thyroid tissue-derived stromal cells, respectively (Figure 3A,B). In contrast, the expression levels of pNF-kB (NF-kB signaling activity indicator) and pSmad3 (TGF-β signaling activity indicator) were not altered in the CSCs that were transwell cocultured with PDX or patient primary tumor stromal cells as compared with the respective controls (Figure 3C,D). Altogether, these observations demonstrated the capacity of tumor stromal cells to enhance JAK/STAT3 and PI3K/Akt signaling in CSCs, suggesting that JAK/STAT3 and PI3K/Akt signaling pathways might be engaged in the functioning of tumor stromal cells on PTC CSCs, while NF-kB and TGF-β signaling were unlikely to have contributed to the tumor stromal cells’ effect, as shown in Figure 1 and Figure 2, on PTC CSCs.

### 2.3. PI3K/Akt and JAK/STAT3 Signaling Engaged in the Promoting Effect of Tumor Stromal Cells on CSC Sphere Formation and Clonal Growth

**Figure 4:**
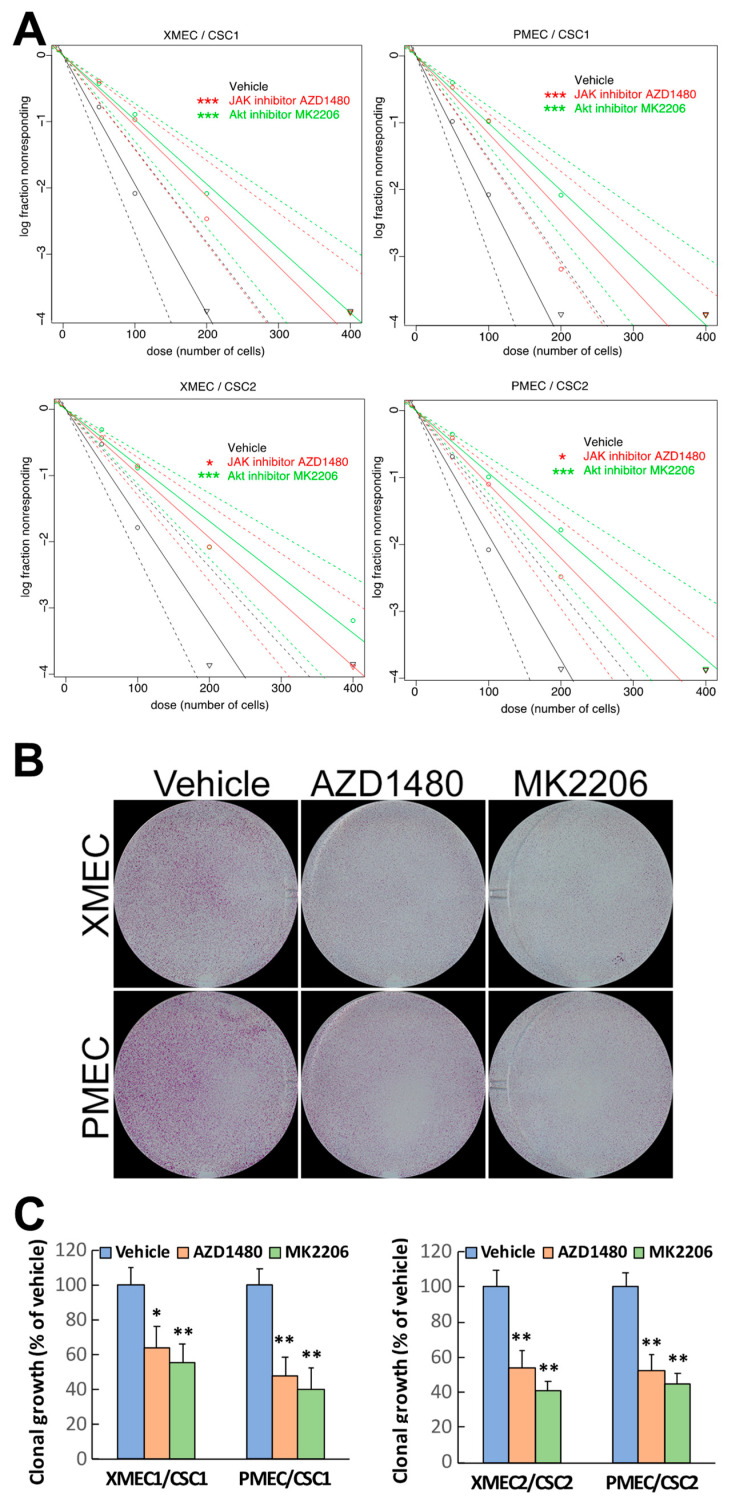
PI3K/Akt and JAK/STAT3 signaling engaged in the effects of tumor stromal cells on CSCs. (**A**) The blockades of PI3K/Akt and JAK/STAT3 signaling reduced the promoting effects of patient-derived xenograft tumor stromal cells (left) and patient primary tumor stromal cells (right) on CSC1 sphere formation. (**B**) The blockades of PI3K/Akt and JAK/STAT3 signaling decreased the enhancement of patient-derived xenograft tumor stromal cells (top panel) and patient primary tumor stromal cells (bottom panel) on CSC1 clonal growth. (**C**) Quantitative analyses of the effects of PIK3/Akt and JAK/STAT3 signaling blockade on the tumor stromal cells enhanced CSC clonal growth. Xenograft tumor microenvironment stromal cell culture (XMEC); patient primary tumor microenvironment stromal cell culture (PMEC). * *p* < 0.05, ** *p* < 0.01, *** *p* < 0.001.

The above data demonstrated that tumor stromal cells were able to activate the PI3K/Akt and JAK/STAT3 signaling and to alter the cellular aggressiveness of PTC CSCs. We subsequently investigated whether the PI3K/Akt and JAK/STAT3 signaling had contributed to the alterations of CSC cellular behaviors. We initially tested the influence of blocking the PI3K/Akt and JAK/STAT3 signaling activity on CSC’s sphere-formation capacity. MK2206 (Akt specific inhibitor) or AZD1480 (JAK specific inhibitor) was added into the CSC-seeded bottom chambers of the transwell cocultures during the initiation of CSC sphere-formation assay. In parallel, the vehicle used for diluting the specific inhibitors was added in the same way to serve as control. We found that blocking PI3K/Akt signaling significantly reduced the CSC sphere-formation activity (Figure 4A). MK2206 reduced both CSC1 and CSC2 cell’s sphere formation compared with the vehicle controls in the transwell cocultures containing tumor stromal cells derived from different CSC generated PDX (Figure 4A, upper panel). In addition, MK2206 was also able to decrease the CSC’s sphere-formation activities in the transwell cocultures containing patient primary tumor stromal cells compared with the vehicle controls (Figure 4A, lower panel). To confirm the involvement of the PI3K/Akt pathway in the alterations of CSC behaviors, we tested a PI3K inhibitor PI-103 in the above sphere-formation assay. PI-103 produced an effect comparable to that obtained in the MK2206 experiments, suggesting that the PI3K/Akt, not PI3K-independent, pathway mediated the Akt signaling activation. Moreover, it was discovered that blocking JAK/STAT3 signaling caused significant reductions of CSC sphere formation in the transwell cocultures seeded with PDX tumor stromal cells in comparison with the vehicle treatment (Figure 4A, upper panel). AZD1480 treatment also decreased CSC sphere formation in patient primary tumor stromal cell-seeded transwell cocultures compared with the vehicle treatment (Figure 4A, lower panel). It is of note that blocking Akt signaling had a higher efficacy in reducing CSC sphere formation than did blocking JAK/STAT3 signaling (Figure 4A). These observations were reproducible in a series of transwell cocultures that contained either PDX or patient primary tumor stromal cells together with the CSCs of different patients, respectively.

Next, we tested whether blocking these signaling pathways has an influence on PTC CSC clonal growth. We observed that blocking either PI3K/Akt or JAK/STAT3 signaling reduced both CSC1 and CSC2 cell clonal growth in the cocultures containing PDX tumor stromal cells (Figure 4B, Supplementary Figure S2). Similar results were obtained in the cocultures containing patient primary tumor stromal cells (Figure 4B, Supplementary Figure S2). Interestingly, the blockade of PI3K/Akt signaling had a stronger inhibiting effect on CSC clonal growth than did the blockade of JAK/STAT3 signaling (Figure 4B,C). These results were consistent with the observations acquired in the sphere-formation assays, which revealed a higher efficacy with blocking PI3K/Akt than blocking JAK/STAT3 signaling in reducing CSC sphere-formation activity (see Figure 4A). Altogether, the above findings demonstrated that both PI3K/Akt and JAK/STAT3 signaling pathways contributed to the promoting effects of tumor stromal cells on PTC CSC sphere formation and cloning growth, and that the PI3K/Akt pathway had greater contributions than did the JAK/STAT3 pathway.

### 2.4. PI3K/Akt Signaling Pathway Contributed to the Enhanced CSC Invasion Induced by Tumor Stromal Cells

**Figure 5:**
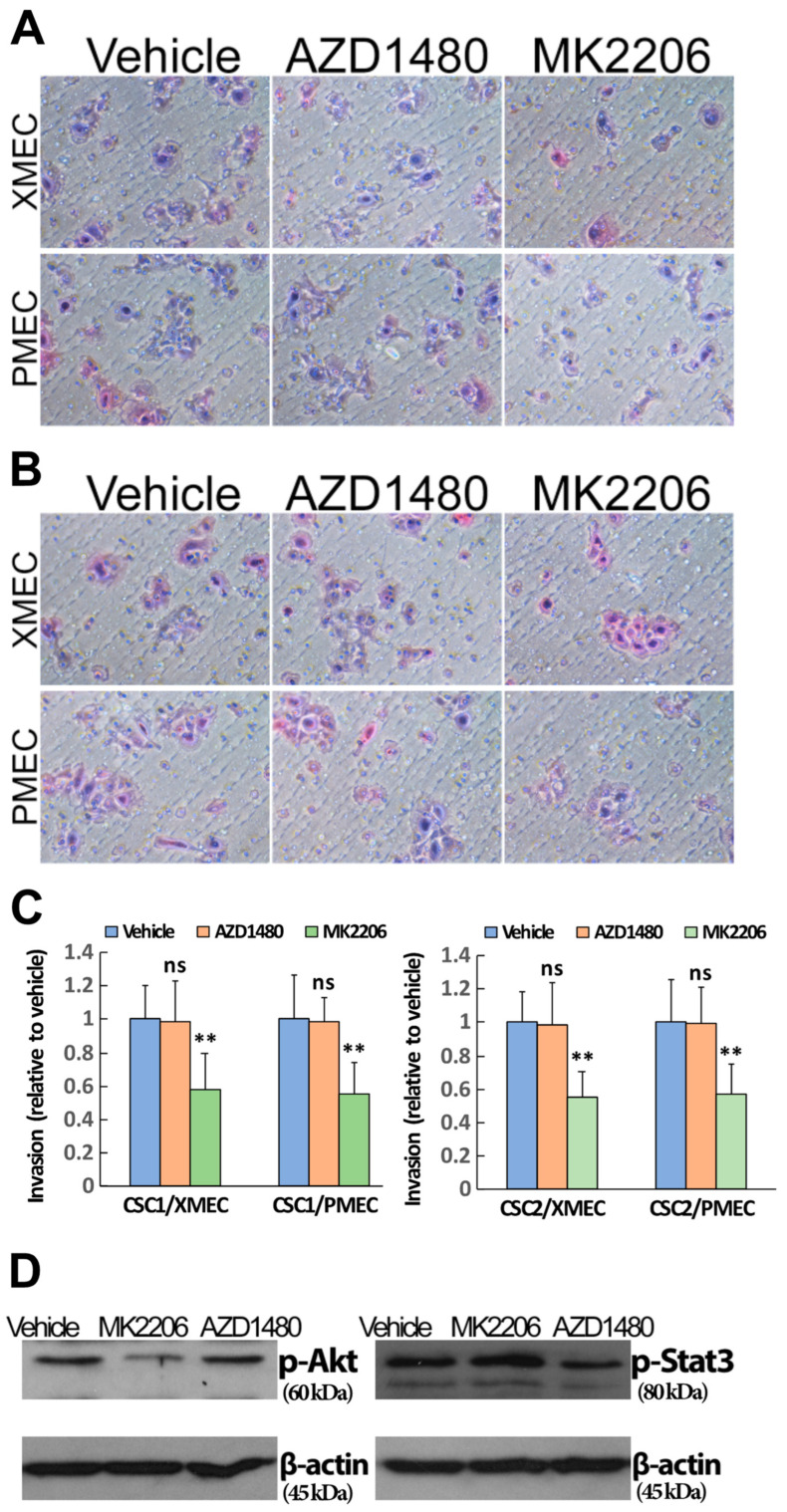
PI3K/Akt signaling pathway contributed to the effects of tumor stromal cells on CSC invasiveness. (**A**) The blockade of PI3K/Akt signaling reduced the enhancement of CSC cell invasion induced by either patient-derived xenograft tumor stromal cells (top panel) or patient primary tumor stromal cells (bottom panel). The blockade of JAK/STAT3 signaling did not affect the cell invasion. (**B**) The effects of PI3K/Akt and JAK/STAT3 signaling blockade on CSC invasive activity were reproducible in a different patient-derived CSCs. (**C**) Quantitative analyses showed significant effects of PI3K/Akt signaling blockade on the enhanced CSC invasion. The JAK/STAT3 signaling blockade did not significantly affect the enhancement of CSC invasion. (**D**) Blocking PI3K/Akt signaling decreased the pAkt expression compared with the vehicle and the blockade of JAK/STAT3 signaling (left panel). Blocking JAK/STAT3 signaling decreased the pStat3 expression compared with the vehicle and the blockade of PI3K/Akt signaling (right panel). ** *p* < 0.01; ns, not significant. Scale bars: 100 µm.

Tumor stromal cells enhanced the cell invasion and the cell signaling activities of both PI3K/Akt and JAK/STAT3 pathways in patient PTC-derived CSCs (see Figure 2 and Figure 3). Given these observations, we wanted to determine whether the enhanced cell signaling activities were relevant to the enhancement of the cell invasion. To this end, we investigated the potential influences of PI3K/Akt and JAK/STAT3 signaling blockade on CSC invasive behavior. Blocking PI3K/Akt signaling with MK2206 treatment significantly diminished CSC invasion toward PDX tumor stromal cells compared with the vehicle treatment (Figure 5A, upper panel). The influence of MK2206 on CSC invasive activity was also observed when patient primary tumor stromal cells were involved in the invasion assays (Figure 5A, lower panel). In contrast, blocking JAK/STAT3 signaling with AZD1480 treatment barely had effects on the CSC invasive activity regardless of whether PDX or patient primary tumor stromal cells were involved in the invasion assays (Figure 5A,C). To validate the reliability of these results, we repeated the above experiments with another two CSC samples isolated from different PTC patients. It was revealed that comparable results were obtained when the different CSC samples were subjected to the analyses (Figure 5B,C).

To validate that AZD1480′s inability to influence CSC invasion was not an artifact derived from the inadequate blockade of JAK/STAT3 signaling activity, we analyzed the pAkt and pSTAT3 expression in the MK2206, AZD1480 and vehicle-treated CSCs within the invasion assays by Western blotting. It was found that MK2206 and AZD1480 treatment effectively reduced CSC pAkt and pSTAT3 expression, respectively, within the invasion assays that were involved in either PDX or patient primary tumor stromal cells (Figure 5D). Similar profiles of pAkt and pSTAT3 expression were obtained when another two different CSC samples were subjected to the invasion analyses. Altogether, the above data demonstrated that PI3K/Akt signaling contributed to the tumor stromal-cell-induced enhancement of PTC CSC invasion, while the JAK/STAT3 signaling was not relevant to the invasion enhancement.

### 2.5. Inhibiting Akt Signaling Activity Represents a Therapeutic Strategy to Target PTC Tumor Progression

**Figure 6:**
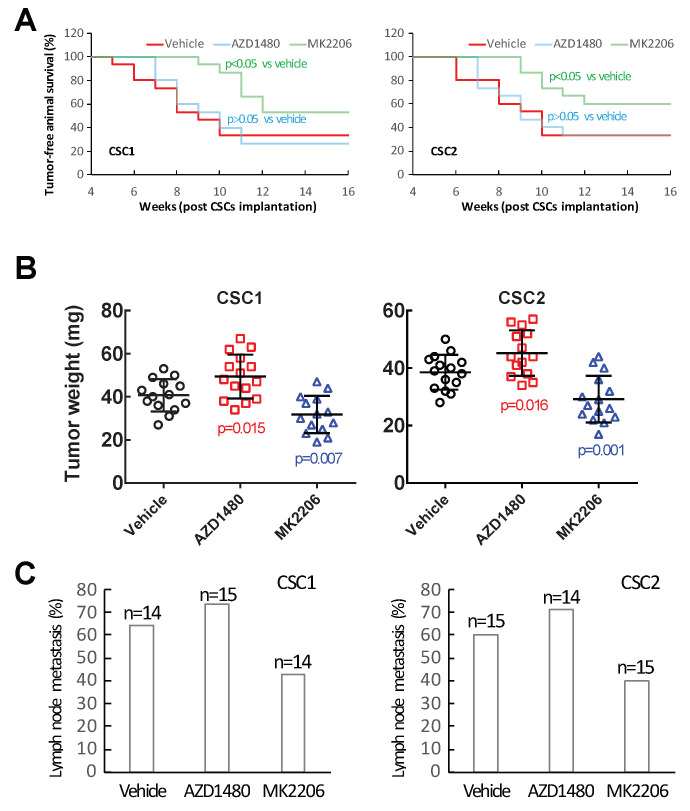
Inhibition of PI3K/Akt signaling reduced CSC tumorigenesis and tumor progression. (**A**) MS2206 treatment decreased CSC tumor formation efficiency compared with vehicle and AZD1480 treatment in subcutaneous xenograft tumor models. (**B**,**C**) MS2206 treatment reduced PTC tumor growth (**B**) and lymph node metastasis (**C**) compared with vehicle and AZD1480 treatment in orthotopic xenograft tumor models.

Given that PI3K/Akt and JAK/STAT3 signaling pathways engaged in PTC tumor stromal-cell-enhanced CSC aggressive behaviors in vitro, we investigated whether these signaling pathways were relevant to PTC tumorigenesis and tumor progression. Patient PTC-derived CSCs together with PDX tumor stromal cells were subcutaneously implanted into the flanks of NOD/SCID mice. The implanted mice were subsequently given either MK2206, AZD1480 or vehicle treatment. Tumor formations in the animals were monitored during the different treatments. Inhibiting Akt signaling significantly reduced tumor formation efficiencies in comparison with the vehicle treatment (Figure 6A). In contrast, inhibiting JAK/STAT3 signaling did not significantly affect the efficiencies of tumor formation (Figure 6A), although the tumor latency was slightly altered compared with the vehicle-treated controls. To confirm these observations, we performed the xenograft implantation with another two CSC samples, CSC2 and CSC5, that were derived from different PTC patients, and subjected the implanted mice to the same treatments described above. We found that comparable results were obtained when the CSC2 and CSC5 samples were tested in the tumorigenic assays (Figure 6A, Supplementary Figure S3A). These data revealed that inhibiting Akt signaling was able to reduce the capacity of CSC tumor initiation while inhibiting JAK/STAT3 signaling did not have a significant impact on the CSC tumorigenic potential.

**Figure 7:**
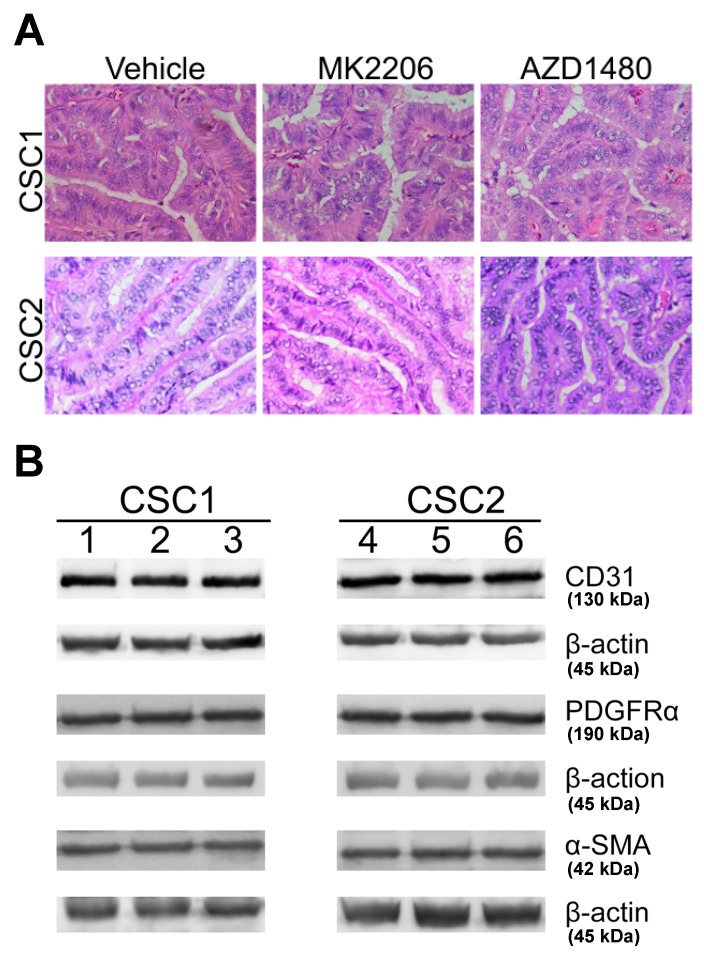
Inhibition of PI3K/Akt and JAK/STAT3 signaling had no detectable influence on tumor histology and the expression of the fibroblast and vasculature genes. (**A**) Tumor histology was not altered after MS2206 (middle column) and AZD1480 (right column) treatment compared with the vehicle (left column) treatment. (**B**) Vascular endothelial cell CD31 expression did not show alterations after the MS2206 (lane 2, 5) and AZD1480 (lane 3, 6) treatment compared with the vehicle (lane 1, 4) treatment; cancer-associated fibroblast markers PDGFR-⍺ and ⍺-SMA expression did not show alterations after the MS2206 (lane 2, 5) and the AZD1480 (lane 3, 6) treatment compared with the vehicle (lane 1, 4) treatment. Scale bars: 100 µm.

Next, we wanted to assess whether PI3K/Akt and JAK/STAT3 signaling inhibition could function as potential treatments targeting PTC tumor progression. To this end, we established an orthotopic xenograft tumor model by implanting CSCs into the right thyroid of NOD/SCID mice. Four weeks after the implantation, which allowed the orthotopic tumor formation [53], the tumor-bearing animals began receiving either MK2206, AZD1480 or vehicle treatment. Upon completion of the treatments, animals were evaluated for signs of orthotopic primary tumors. The MK2206 treatment significantly reduced the orthotopic tumor volumes compared to the vehicle treatment (Figure 6B). Interestingly, the AZD1480 treatment increased the tumor volumes in comparison with both the vehicle and the MK2206 treatment (Figure 6B). These results were reproducible in three different CSC-sample-derived orthotopic tumors (Figure 6B, Supplementary Figure S3B), suggesting that the PI3K/Akt and the JAK/STAT3 signaling played different roles in the process of PTC tumor development. To further explore possible differences between the inhibition of PI3K/Akt and JAK/STAT3 signaling, we examined the cervical lymph nodes of the treated animals for potential signs of metastasis. Inhibiting PI3K/Akt signaling resulted in fewer animals with lymph node metastasis compared with the vehicle treatment (Figure 6C, Supplementary Figure S3B). Among the animals that had developed lymph node metastasis, fewer metastatic nodes were found in the MK2206-treated mice (1–2 nodes/mouse) compared with the vehicle-treated ones (2–3 nodes/mouse; MK2206 vs. vehicle, *p* < 0.05). In contrast, inhibiting JAK/STAT3 signaling as treatment ended with more animals having lymph node metastasis in comparison with the inhibition of PI3K/Akt signaling and the vehicle treatment (Figure 6C, Supplementary Figure S3B). There were no significant differences observed in the metastatic lymph node numbers among the animals that received either AZD1480 or vehicle treatment. Furthermore, we investigated whether the PI3K/Akt and JAK/STAT3 signaling inhibition had any influence on tumor histological features. It was found that tumors from the MK2206, AZD1480 and vehicle treated animals showed comparable histology (Figure 7A). In addition, Western blotting analysis of tumor tissues for vasculature marker CD31 expression did not identify differences among the different treatment groups (Figure 7B). Similarly, the TME fibroblast markers, PDGFR⍺ and ⍺-SMA [84,85], showed comparable expression levels between the tumors that received different treatments (Figure 7B). Altogether, the above results demonstrated that inhibiting PI3K/Akt signaling had therapeutic benefits on PTC primary tumor development and metastasis formation, while inhibiting JAK/STAT3 signaling had no beneficial effects on both the primary tumors and the metastases in an orthotopic xenograft model. In addition, no alterations in tumor vasculature, histology and cancer associated fibroblast cell population were identified after the disruptions of the two respective signaling pathways.

To further explore the potential clinical relevance of targeting the PI3K/Akt signaling in thyroid cancer, we directly examined the pAkt expression by Western blotting in a cohort of surgically resected patient PTC specimens and non-tumor thyroid tissues. In this clinical cohort, 71 patients were recruited by selecting classical PTC as well as the aggressive histological subtypes of the tall cell variant, columnar cell variant, diffuse sclerosing variant, and solid variant. None of the patients had developed recurrent diseases after their initial surgical treatment by the end of current study. It was found that the tumor tissues from patients with lymph node metastasis showed the majority cases as having significant pAkt expression (Table 1). On the other hand, the tumor tissues from patients without lymph node metastasis showed a small proportion of the cases had elevation of pAkt expression (Table 1). All the pAkt-elevated tumor specimens from patients with lymph node metastasis also demonstrated the mutation of BRAF^V600E^, while those pAkt-elevated specimens from patients without lymph node metastasis were BRAF^V600E^ mutation positive only in a proportion of the cases (Table 1).

In sum, our findings implicated that inhibiting Akt signaling represents a potential therapeutic modality to target tumor progression in PTC patients who present with aggressive disease and elevated PI3K/Akt signaling pathway activity. 

## 3. Discussion

The individual components that contribute to the TME, including malignant cancer cells, non-malignant infiltrating stromal cells, and extracellular matrix proteins, function in concert to establish an essential niche that is permissive for tumorigenesis [86]. While various studies have investigated the involvement of individual cell types, such as fibroblasts, immune cells, or cancer cells in the development of tumors, few studies have explored the coordinated effects among the multiple cell types within the TME and how these cells function together to influence tumor growth. In this study, we investigated the coordination among stromal and tumor cells of thyroid cancer. Using primary cells of clinical specimens and using PDX models, we elucidated the effects and mechanisms of non-malignant cells affecting cancer cells, and explored a potential therapeutic strategy for targeting thyroid cancer progression.

It has been reported that the activation of the signaling cascades associated with BRAF and PTEN mutations, two major alterations frequently found in advanced PTCs, results in thyrocytes in the development of PTC with a fibrotic and reactive tumor stroma in a transgenic mouse model [87]. In this transgenic cancer model, fibroblasts were recruited to the TME by tumor cells. Within the TME, fibroblasts were activated, by going through a series of biological processes and causing a stiffer matrix, to augment tumor cell invasion and promote tumor progression [87]. Consistent with the findings derived from transgenic mouse tumors, our data demonstrated that, in human thyroid cancer, tumor stromal cells exerted their function to enhance CSC aggressive behaviors, which were mediated by Akt signaling activity elevation in CSCs. Our findings revealed potential mechanisms that contribute to the effects of tumor stroma cells in promoting thyroid cancer aggressiveness.

In the current study, both PDX and patient primary tumor stromal cells demonstrated the capacity to enhance thyroid CSC aggressive behaviors. These data suggested the reliability of using PDX tumors in elucidating the influence of tumor stromal cells on cancer cell behaviors. It is worth noting that patient primary tumor stromal cells showed higher capacities than did the PDX tumor stromal cells in promoting CSC sphere formation and clonal growth. These variations in capacity were likely due to differences between the PDX and the patient primary tumor stroma. For example, the immune cells and other cells that were either absent in PDX tumors or distinct between PDX and patient primary tumors. How the cells that contributed to the varied capacities exert their influence on CSC behaviors is currently under investigation in another related project.

Our in vitro study revealed the differences between PI3K/Akt and JAK/STAT3 pathway in contributing to tumor stromal cells’ influence on thyroid CSCs. Such differences were also evidenced in xenograft models as the tumor growth and metastasis formation increased upon JAK/STAT3 signaling inhibition while they decreased with Akt signaling inhibition. The functions of JAK/STAT3 signaling discovered in our study were consistent with a previous finding that revealed the roles of JAK/STAT3 signaling in promoting thyroid cancer development in a transgenic tumor model [88]. Thus, our data suggested that although JAK/STAT3 signaling could be upregulated by TME stromal cells and could influence CSC behaviors, targeting the signaling will be unlikely to be beneficial to thyroid cancer patients. In contrast, inhibiting Akt activity may serve as a therapeutic modality to target thyroid cancer disease progression.

Akt signaling inhibition as a treatment for pre-formed cancer had no significant influence on tumor histology, vasculature and fibroblast properties. These findings implied that targeting Akt signaling activity in thyroid cancer had no obvious effects on tumor pathological features while producing therapeutic benefits, including reducing tumor growth and decreasing metastasis formation. It was previously reported in mouse models that the biomechanical properties of tumor-associated extracellular matrix can have strong influences on cancer cell behaviors [89], and that the activation of the PI3K/Akt pathway can lead to TME matrix stiffness alternations [87]. Although additional evidence is needed to confirm whether the inhibition of Akt signaling in our experiments impacted TME matrix stiffness, our data on tumor histology and major stromal component gene expression did not support a clear involvement of TME matrix alternation in the observed therapeutic benefits derived from Akt signaling inhibition.

Thyroid cancers in advanced forms are associated with mutations in the MAPK pathway and additional genetic alterations, which can result in constitutive PI3K/Akt pathway activation [90]. Our data revealed the increases in CSC Akt activity that were correlated with enhanced CSC aggressive cellular behaviors. Interestingly, Akt activity in CSCs was also detected, although at comparatively low levels, in conditions without association with tumor stromal cells. These observations suggested the existence of Akt signaling activity in thyroid CSCs that could be enhanced when the cells were interacting with tumor stromal cells. Thus, our findings implicated that inhibiting Akt signaling as a therapy will likely target the PI3K/Akt pathway activation derived from either genetic aberration, tumor stromal cell stimulation, or both together.

PTCs that developed lymph node metastases most likely have elevated Akt activity as well as BRAF^V600E^ mutation as revealed in the present study. Since BRAF mutations were found to be associated with fibrotic TME and unfavorable clinical outcomes in PTC patients [87,91,92,93], blocking Akt activity as investigated in the current study might represent an effective strategy that can attenuate the PI3K/Akt signaling in the TME associated with BRAF mutation and metastasis. Such a strategy will likely be able to target thyroid cancer disease progression in the BRAF mutated cases that constitute the majority of aggressive PTCs [92,93].

It is currently been well recognized that the TME is indispensable for tumorigenesis. Meanwhile, most published studies only address the contribution of a singular component of TME to tumor progression. Given that the TME is composed of multiple components, investigations that intend to explore how these components function together to promote tumorigenesis and tumor progression are extremely critical for fully understanding the mechanisms of tumor development and resistance to therapy. Our findings on TME stromal cells functioning on thyroid CSCs provide the proof-of-concept for developing more effective therapeutic strategies in our cancer progression control. The development of such strategies will account for the contribution of stromal cells in clinical condition by targeting aggressive cancer cell activities that are linked with the TME non-tumor elements.

## 4. Materials and Methods

### 4.1. Preparation of Primary Cells from Patient PTC and Non-Tumor Thyroid Tissues

Human PTC and non-tumor thyroid tissues were obtained intraoperatively after informed consent, as approved by the Institutional Review Board at Loma Linda University (IRB #5140014). Tissue specimens were washed in PBS, minced with sterile blades and incubated with collagenase (STEMCELL Technologies, Cambridge, MA, USA) for 1.5 h (h) at 37 °C. After this enzymatic digestion, the sample was filtered through a 40 μm cell strainer and mixed with ammonium chloride solution for 2 min to lyse red blood cells. Single cells were subject to subsequent experiments.

### 4.2. PTC and Non-Tumor Thyroid Tissue-Derived Cells In Vitro Cultures

The prepared PTC cells were plated in serum-free culture (DMEM/F12 medium supplemented with B27, N2, EGF and bFGF (20 ng/mL each), nonessential amino acids (NEAA), sodium pyruvate (SP), and penicillin-streptomycin-amphotericin B (P-S-A)) as described previously for isolation of thyroid CSCs [53]. In the serum-free conditions, cells were growing in low-attachment 6-well plates as spheres. The spheres were collected by gentle centrifugation and dissociated enzymatically with 0.05% trypsin/EDTA. The dissociated cells were passed through a 40 μm mesh filter to obtain single-cell suspensions. The sphere cells isolated in this process represent CSCs as demonstrated in our previous study [53]. Single cells were plated within the lower chambers of transwell cocultures either in low-attachment plates for sphere-formation assay or cell-culture-coated plates for colony growth assays. In some experiments, spheres were collected, dissociated into single-cell suspensions, and then mixed with Matrigel/DMEM/F12 for injection into mice. The prepared non-tumor thyroid cells were plated in serum-containing (RPMI 1640 medium supplemented with 10% fetal bovine serum, NEAA, SP, and P-S-A) conditions growing as a monolayer. Tumor and thyroid stromal cells (from both patients and mice) were obtained by depletion of epithelial cell adhesion molecule positive (EpCAM^+^) cells from the PTC and non-tumor thyroid tissue-derived single cells, respectively, with MicroBeads (Miltenyi Biotec, San Diego, CA, USA) column-based magnetic cell isolation.

### 4.3. Patient Derived Xenograft Tumors

Eight-week-old NOD/SCID mice were obtained from Jackson Laboratory and maintained under specific pathogen-free conditions with the approval of the Institutional Animal Care and Use Committee of Loma Linda University. For orthotopic transplantation, single cells derived from sphere cells were resuspended in Matrigel/DMEM/12 (1:1) into the desired cell doses (10^5^ cells/mouse), and injected into the right thyroid gland that was exposed by surgical procedures and closed by sutures after the injection. Mice were euthanized 20 weeks post-injection and examined for tumor formation and cervical lymph node metastasis. For subcutaneous transplantation, sphere-derived single cells were resuspended in Matrigel/DMEM/12 into the desired cell doses (5 × 10^4^ cells/mouse) together with PTC stromal cells (10^5^ cells/mouse), and injected subcutaneously into the flanks of NOD/SCID mice. The tumor development was measured after the injection. These mice were euthanized at the end of experiments (16 weeks). The morphological features of xenograft tumors were analyzed after hematoxylin-eosin staining of the tissue sections and examined under a microscope. For the Akt inhibitor and JAK inhibitor treatment, MS2206 at 120 mg/kg three times per week for 16 weeks by oral gavage and AZD1480 at 50 mg/kg once a day for 16 weeks by oral gavage [94,95,96]. Both inhibitors were from Selleck Chemicals (Houston, TX, USA).

### 4.4. Sphere Formation and Clonal Growth

For the sphere-formation assay, tumor spheres harvested from primary cultures were dissociated into single cells, and viable cells were seeded into the bottom chambers of the transwell cocultures (low-attachment plate with insert pore size 1.0 μm) at serial cell doses. Tumor or non-tumor thyroid tissue stromal cells were seeded in the top chambers. Two weeks later, the wells of the lower chambers containing spheres were scored, and the number of positive wells was used to calculate the frequency of sphere-forming units using the ELDA software provided by the Walter and Eliza Hall Institute [97] as described previously [53]. Viable cells were determined by trypan blue exclusion analysis. The percentage of trypan-blue-negative cells was used to calculate the frequencies of sphere-forming cells. For the colony growth assay, the bottom chambers of transwell plates were pre-coated with poly-D-lysine before the seeding of sphere-derived single cells. One week later, the bottom chambers were stained with crystal violet solution (0.75% crystal violet, 50% ethanol, 0.25% NaCl, 1.57% formaldehyde). Colonies stained by the crystal violet were identified under microscopy and counted with ImageJ. For the signaling pathway blockade, the Akt inhibitor MK2206 and JAK inhibitor AZD1480 were added into the bottom chambers with the final concentrations as 0.5 and 1 μmol/L, respectively, during the sphere formation and colony growth.

### 4.5. Tumor Cell Invasion Assay

Tumor sphere cell invasion was assessed via Boyden chamber assay with 24-well plate inserts (8.0 μm pore size) pre-coated with standard Matrigel (Corning, Tewksbury, MA, USA). The subsequent detection of invasive cells was performed with either crystal violet or hematoxylin-eosin staining. The bottom chambers were seeded with 0.5–1 × 10^5^ of tumor or non-tumor thyroid tissue-derived stromal cells in serum-containing medium and allowed to grow for 12–24 h before setting up the top insert chambers. The top chambers were seeded with 2.5 × 10^4^ sphere cells resuspended in serum-free CSC culture medium. The sphere cells were allowed to invade for 22 h. Upon the completion of the 22-h invasion, non-invasive cells in the top chamber were removed with cotton swabs, and invasive cells on the insert bottom membrane were fixed in 1% paraformaldehyde and processed for staining. Stained cells were scored under the microscope. Independent invasion experiments were performed in duplicate or triplicate wells. For the signaling pathway blockade, the Akt inhibitor MK2206 and JAK inhibitor AZD1480 were added into the top chambers with the final concentration as 0.5 and 1 μmol/L [95,96,98], respectively, during the sphere cell invasion.

### 4.6. Western Blotting

Tumor sphere cells from the transwell cocultures or Boyden chamber invasion assays and tissues from patient primary tumors or PDX tumors were lysed with Pierce IP lysis buffer containing protease inhibitor (Thermo Fisher Scientific, Waltham, MA, USA). Lysates were quantified with BCA assay kit (Thermo Fisher Scientific). A 10–100 μg quantity of total protein was separated by SDS-PAGE, and protein was transferred onto a polyvinylidene fluoride membrane (Millipore, Burlington, MA, USA) [99]. Specific antibodies to phospho-Stat3 (Tyr705), phospho-Akt (Ser473), phospho-NF-kappaB p65 (Ser536), phospho-Smad3 (Ser423/425), CD31, PDGF Receptor α (PDGFRα), and α-smooth muscle actin (α-SMA) (1:1000; all from Cell Signaling, Danvers, MA, USA), and B-Raf (V600E) (1:1000; Thermo Fisher Scientific) were detected using the appropriate secondary horseradish peroxidase-conjugated antibodies, and visualized by an enhanced chemiluminescence detection system (Bio-Rad, Hercules, CA, USA). β–actin was detected using horseradish peroxidase-conjugated anti-β-actin antibody (1:10,000, Cell Signaling) and served as protein loading controls.

### 4.7. Statistics

Limiting-dilution analyses (LDAs), for frequency determinations of sphere-forming cells and the corresponding *p*-values, were performed using ELDA software, which took into account whether the assumptions for LDA were met [97]. The data and error bars reported the mean ± SEM. *: *p* < 0.05, **: *p* < 0.01 and ***: *p* < 0.001. For comparisons of colony numbers, invaded cell numbers, xenograft tumor weights, and metastasis rates, a two-tailed Student’s *t* test was performed between two groups and a difference was considered statistically significant with *p* < 0.05.

## 5. Conclusions

Overall, this study identifies the effects and underlying mechanisms of TME stromal cells in promoting thyroid CSC aggressiveness, provides evidence suggesting that TME Akt signaling plays critical roles in driving thyroid tumor progression, and demonstrates the relevance of targeting TME Akt signaling in thyroid cancer patients. Thus, these findings may assist clinicians to design suitable therapeutic strategies for aggressive cases of thyroid carcinoma.

## Figures and Tables

**Table 1:** The pAkt elevation is relevant to lymph node metastasis and BRAF mutation status.

Metastasis Status	PTC Tested (Cases)	pAkt Expression ↑ (Cases)	pAkt Expression ↑ and BRAF^V600E^ (Cases)
Lymph node ^+^	26	25	25
Lymph node ^−^	45	11	6

↑: expression upregulation; −: metastasis negative; +: metastasis positive.

## Data Availability

No new data were created or analyzed in this study. Data sharing is not applicable to this article.

## Supplementary Materials

The supporting information can be downloaded at: https://www.mdpi.com/article/10.3390/ijms24065471/s1.
