# Supplementary material for: Targeting Tumor Microenvironment Akt Signaling Represents a Potential Therapeutic Strategy for Aggressive Thyroid Cancer
Source: Int J Mol Sci. 2023 Mar 13;24(6):5471. doi: 10.3390/ijms24065471 (PMC10049397; doi:10.3390/ijms24065471)
Supplement: Supplementary file 1 [file ijms-24-05471-s001.zip › ijms-2242955-supplementary.pdf]

Supplementary Figure S1

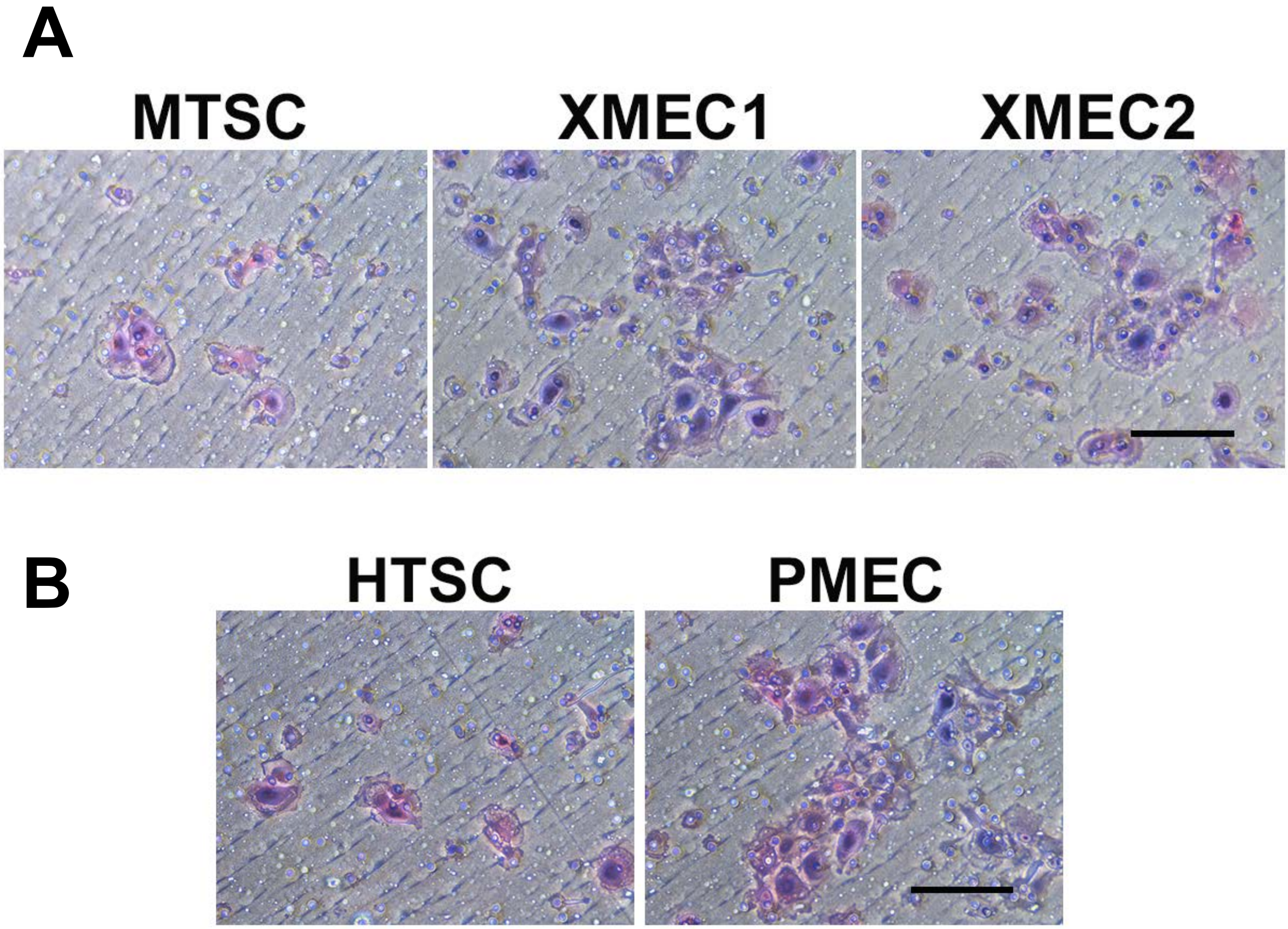

Supplementary Figure S1. **PTC tumor stromal cells enhanced CSC invasiveness. A** Patient-derived xenograft tumor stromal cells increased CSC2 cell invasive activities in Boyden chamber invasion assays. **B** Patient primary tumor stromal cells increased CSC2 cell invasive activities. Mouse normal thyroid stromal cell culture (MTSC), xenograft tumor microenvironment stromal cell culture (XMEC), patient non-tumor thyroid stromal cell culture (HTSC), patient primary tumor microenvironment stromal cell culture (PMEC). Scale bar: 100 μm

Supplementary Figure S2

CSC2

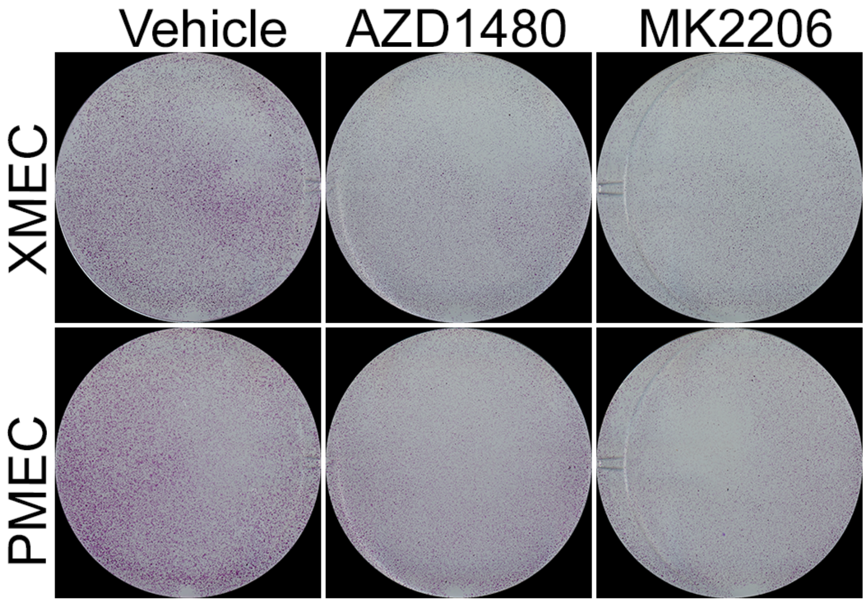

Supplementary Figure S2. **PI3K/Akt and JAK/STAT3 signaling engaged in the effects of tumor stromal cells on CSC clonal growth**. The blockades of PI3K/Akt and JAK/STAT3 signaling decreased the enhancement of patient-derived xenograft tumor stromal cells (top panel) and patient primary tumor stromal cells (bottom panel) on CSC2 clonal growth. Xenograft tumor microenvironment stromal cell culture (XMEC); patient primary tumor microenvironment stromal cell culture (PMEC).

Supplementary Figure S3

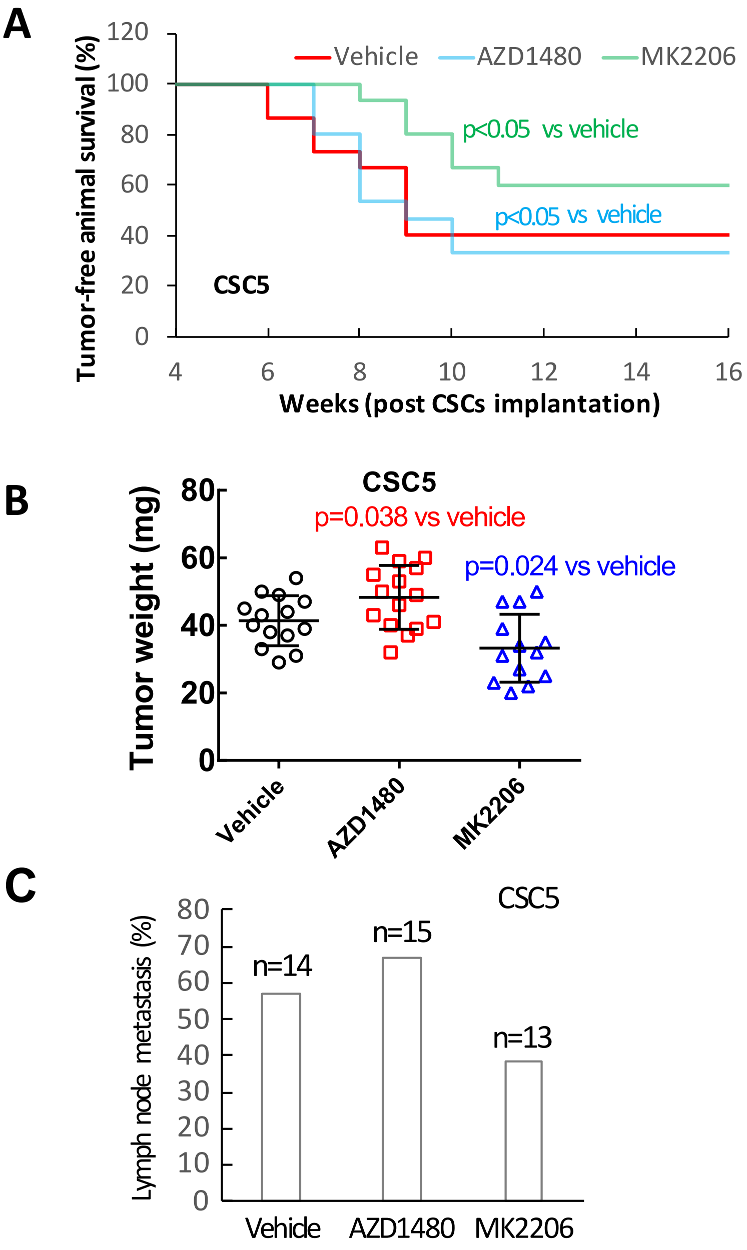

Supplementary Figure S3. **Inhibition of PI3K/Akt signaling reduced CSC tumorigenesis and tumor progression. A** Akt inhibitor MS2206 treatment decreased CSC tumor formation efficiency compared with vehicle and JAK/STAT3 signaling inhibitor AZD1480 treatment in a subcutaneous xenograft tumor model. **B, C** MS2206 treatment reduced PTC tumor growth (B) and lymph node metastasis (C) compared with vehicle and AZD1480 treatment in an orthotopic xenograft tumor model.
